# Macrophage Polarization in Cardiac Tissue Repair Following Myocardial Infarction

**DOI:** 10.3390/ijms22052715

**Published:** 2021-03-08

**Authors:** Yevgeniy Kim, Sanzhar Nurakhayev, Ayan Nurkesh, Zharylkasyn Zharkinbekov, Arman Saparov

**Affiliations:** Department of Medicine, School of Medicine, Nazarbayev University, Nur-Sultan 010000, Kazakhstan; yevgeniy.kim@nu.edu.kz (Y.K.); sanzhar.nurakhayev@nu.edu.kz (S.N.); ayan.nurkesh@nu.edu.kz (A.N.); zharylkasyn.zharkinbekov@nu.edu.kz (Z.Z.)

**Keywords:** macrophages, cardiac tissue repair, macrophage polarization, myocardial infarction

## Abstract

Cardiovascular disease is the leading cause of mortality and morbidity around the globe, creating a substantial socio-economic burden as a result. Myocardial infarction is a significant contributor to the detrimental impact of cardiovascular disease. The death of cardiomyocytes following myocardial infarction causes an immune response which leads to further destruction of tissue, and subsequently, results in the formation of non-contractile scar tissue. Macrophages have been recognized as important regulators and participants of inflammation and fibrosis following myocardial infarction. Macrophages are generally classified into two distinct groups, namely, classically activated, or M1 macrophages, and alternatively activated, or M2 macrophages. The phenotypic profile of cardiac macrophages, however, is much more diverse and should not be reduced to these two subsets. In this review, we describe the phenotypes and functions of macrophages which are present in the healthy, as well as the infarcted heart, and analyze them with respect to M1 and M2 polarization states. Furthermore, we discuss therapeutic strategies which utilize macrophage polarization towards an anti-inflammatory or reparative phenotype for the treatment of myocardial infarction.

## 1. Introduction

Cardiovascular disease (CVD) is the leading cause of mortality worldwide, with a great social and economic impact on public health [1]. In 2019, CVD accounted for 18.6 million deaths, which corresponded with a 6.5 million increase since 1990 [2]. Moreover, it is predicted that the mortality rate will increase up to 23 million per year by 2030 [3]. The quality of life after CVD is also affected, and years of life with disability among survivors doubled from 17.7 million in 1990 to 34.4 million in 2019 [2]. The financial burden has also increased over the years, with the total cost of CVD reaching 318 million USD in 2015 in the United States alone [3]. In addition, cost is projected to increase significantly for adults over 65 and could reach 1.1 trillion USD by 2035 [4,5]. Ischemic heart disease (IHD) and myocardial infarction (MI) are major contributors to CVD mortality [6]. In 2016 alone, IHD claimed the lives of almost 9 million people worldwide [6]. IHD may lead to the development of MI, which occurs when blood supply decreases or ceases to a part of the heart, causing necrosis of myocardial cells [7]. Death of cardiomyocytes and subsequent inflammation lead to fibrosis, cardiac scarring and adverse remodeling, which negatively impacts the regeneration of the myocardium and could potentially result in heart failure and death [8].

Immune response plays a crucial role in the pathogenesis of MI [9,10]. In response to ischemia, resident macrophages and cardiomyocytes release pro-inflammatory cytokines and chemokines, namely, interleukin-1 (IL-1), interleukin-6 (IL-6), tumor necrosis factor (TNF) and CC-chemokine ligand 2 (CCL2), while resident mast cells secrete pre-formed TNF-α [11,12]. In addition, cardiac fibroblasts produce hematopoietic growth factors such as granulocyte-macrophage colony-stimulating factor (GM-CSF), and endothelial cells of coronary blood vessels upregulate the expression of vascular cell adhesion molecule 1 (VCAM-1) and selectins [11]. Moreover, the death of cardiomyocytes leads to the release of damage-associated molecular patterns (DAMPs), such as heat shock proteins, nuclear chromatin-binding protein high mobility group box 1, low molecular hyaluronic acid, fibronectin fragments, cardiac myosin, mitochondrial DNA and circulating extracellular RNA molecules [13]. DAMPs are recognized by pattern recognition receptors expressed on neutrophils, macrophages, dendritic cells and other cells of the immune system [13]. All of the aforementioned events result in the massive influx of circulating monocytes and neutrophils into the infarcted tissue [11]. Neutrophils and monocytes migrate to the site of infarction to engulf cellular debris and release matrix metalloproteinases (MMPs), proteolytic enzymes and pro-inflammatory cytokines such as IL-1, TNF and IL-6 [11,14]. MMPs and proteases degrade the extracellular matrix (ECM) and cleave the chemokine C-X-C motif ligands 1, 5 and 8 (CXCL1, CXCL5 and CXCL8), thereby increasing their chemotactic activity [15]. In addition, neutrophils at the site of injury secrete CCL3 and CCL4, which bind to the C-C Motif chemokine receptor 1 (CCR1) expressed on classical monocytes, as well as upregulate the expression of CCL2 and VCAM on endothelial cells [15]. These changes activate endothelial cells and cardiomyocytes, as well as attract more neutrophils and monocytes, resulting in further amplification of inflammation [11,15]. By day 3, neutrophils start to undergo apoptosis and some of them begin to polarize towards anti-inflammatory phenotypes that are able to produce fibrinogen and fibronectin [14].

Resident macrophages play a very important role in the initiation, development and resolution of the immune response following MI [16]. There are four populations of residential macrophages in the heart based on their expression of chemokine receptor type 2 (CCR2) and other markers: T-cell immunoglobulin and mucin domain containing 4+ (TIMD4+) Lymphatic vessel endothelial hyaluronan receptor 1+ (LYVE1+) MHC-II^lo^CCR2−, which is not repopulated by circulating monocytes, TIMD4−LYVE1−MHC-II^hi^CCR2−, which is partially repopulated by circulating monocytes, and two CCR2+MHC-II^hi^ subsets, which are completely repopulated by circulating monocytes [17]. Resident CCR2+ macrophages become activated by tissue injury via Myd88-dependent pathway and produce cytokines and chemokines that attract and activate neutrophils and monocytes, whereas CCR2- macrophages suppress monocyte migration [18]. Three days after MI, millions of neutrophils and monocytes migrate to the infarcted heart and further drive the inflammatory response [19]. The attracted monocytes express high amounts of lymphocyte antigen 6C (Ly6C) and differentiate into macrophages with high phagocytic and proteolytic activities, and produce IL-1, IL-6 and TNF [19,20]. Pericardial Gata6+ macrophages are another subtype that is involved in the immune response after MI [21]. Studies on GATA3 expression in macrophages demonstrated that only a few, if any, GATA3+ cells were present in the left ventricle of healthy animals, but their number significantly increased following MI [22]. Deletion of GATA3 in the myeloid cells preserved heart function by reducing fibrosis and scar area, as well as improving contractile function [22]. It was proposed that targeting GATA3+ macrophages could be utilized for age-related diseases associated with fibrosis [23]. Approximately on day 4, reparative Ly6C^low^ macrophages start to accumulate at the site of injury and secrete vascular endothelial growth factor (VEGF), transforming growth factor-β (TGF-β) and IL-10 to stimulate angiogenesis and fibrosis [19]. One mechanism by which Ly6C^low^ M2-type macrophages improve wound healing is through secretion of MMP-12 and inhibition of neutrophil migration [19]. MMP-12 knockout mice showed an increased neutrophil number with upregulation of MMP-9, reduced fibrosis and the number of myofibroblasts, as well as impaired cardiac function and survival [24]. Importantly, resident cardiac macrophages, rather than monocyte-derived macrophages, are responsible for wound healing and improvement of cardiac function following MI [17]. Thus, depletion of the resident macrophages results in adverse remodeling and worsened cardiac function after MI despite the fact that macrophages derived from monocytes assume a similar phenotype [17].

In this review, we will discuss the macrophage balance following MI. Cardiac macrophages are generally categorized based on their expression of CCR2, Ly6C and MHC-II [25]. In this paper, however, we will consider cardiac macrophages in terms of M1 and M2 phenotypes. M1 macrophages, also known as classically activated and inflammatory macrophages, are involved in phagocytosis and promote inflammation [26]. By contrast, alternatively activated or anti-inflammatory M2 macrophages stimulate tissue repair and regeneration and have pro-fibrotic and pro-angiogenic properties [27]. A stringent balance between M1 and M2 phenotypes is required for a proper resolution of inflammatory response after a disease [28]. This paper reviews the M1 and M2 balance following MI and discusses an opportunity to use polarization of macrophages towards M2 phenotype as a therapeutic strategy for MI treatment.

## 2. Overview of Macrophage Phenotypes and Polarization States

**Figure 1:**
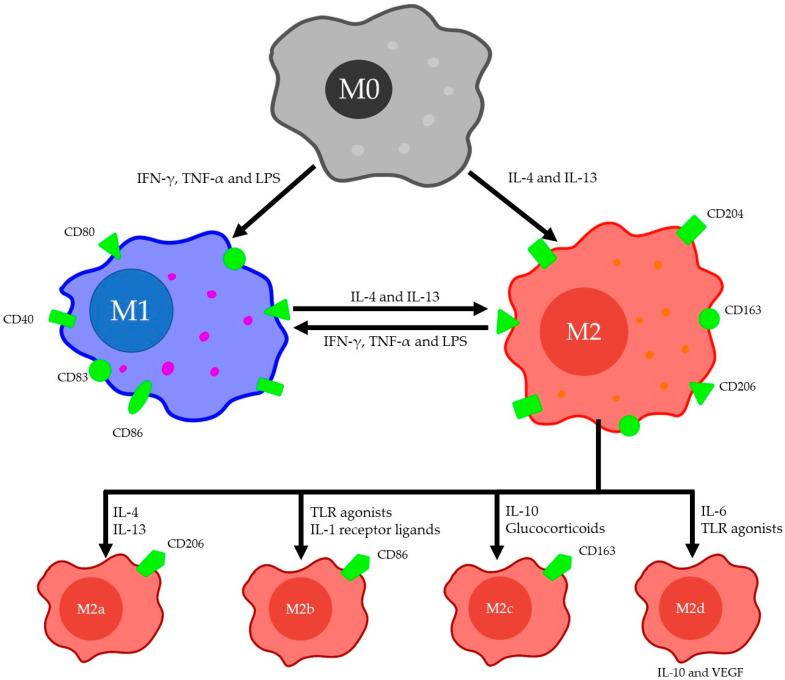
Macrophage Classification. The arrows show the direction and polarization plasticity with inducing factors. Expression of surface markers and production of cytokines/factors by macrophage populations are shown.

Macrophages have long been recognized as important cells not only for the clearance of debris but also for playing a significant role in the tissue regeneration process [29,30,31,32]. Tissue regeneration is highly dependent on the phenotype or polarization state of a macrophage [33]. Macrophages are generally classified into two main groups: classically activated or pro-inflammatory (M1) and alternatively activated or anti-inflammatory macrophages (M2) [26]. They differentiate from monocytes that originate in bone marrow and are derived from the yolk sac during early development to become tissue-resident macrophages [34]. The uniqueness of M1 and M2 macrophages is based on surface markers, function and produced factors. The polarization of monocytes towards M1 phenotype is mediated by type 1 T-helper cell (Th1) cytokines, such as TNF-α and interferon-gamma (IFN-γ), as well as bacterial lipopolysaccharides (LPS). M1 macrophages are differentiated on the basis of CD40, CD80, CD83 and CD86 expression, whereas M2 macrophages express CD204, CD163 and CD206 [35,36,37,38,39]. M1 macrophages secrete pro-inflammatory cytokines such as TNF-α, IL-1α, IL-1β, IL-6, IL-12, IL-23, cyclooxygenase-2 (COX-2) and a low amount of IL-10, all of which contributes to inflammation [40]. In contrast, M2 macrophages are generated by the action of type 2 T-helper cell (Th2) cytokines (IL-4 and IL-13) and produce low amounts of IL-12 and high amounts of IL-10 and TGF-β cytokines that promote anti-inflammatory effects [40]. Interestingly, Gerrick and colleagues reported that conventional macrophage polarization stimuli, e.g., IL-4 and IL-13, were not sufficient in generating macrophages with a complete M2 expression profile and required LPS [41]. Furthermore, the authors identified several novel genes involved in macrophage polarization, namely, that cytochrome b-245 heavy chain (CYBB) was important in M1 polarization, while 7-dehydrocholesterol reductase (DHCR7) was involved in M2 activation. Additionally, specific factors can cause M2 macrophages differentiation into four subtypes—M2a, M2b, M2c and M2d—each of which produces alternative sets of cytokines (Figure 1). Specific factors include, but are not limited to, IL-4 or IL-13 for M2a, toll-like receptor (TLR) agonists or IL-1 receptor ligands for M2b, IL-10 or glucocorticoids for M2c and IL-6 or Toll-like receptor (TLR) agonists for M2d [42,43]. M2a macrophages, or wound healing M2, are characterized by an enhanced expression of CD206, CCL2, CCL17, CCL22 and CCL24 [43]. M2a macrophages play a significant role in ECM formation, as well as tissue repair and restoration, by secreting TGF-β, fibronectin, β IG-H3 and factor VIII subunit A. M2b macrophages are characterized by CCL1, tumor necrosis factor superfamily member 14 (TNFSF14) and CD86 markers [42]. They are known to control the strength of the immune response by their ability to secrete pro-inflammatory cytokines (IL-1β, IL-6 and TNF-α) as well as an anti-inflammatory cytokine IL-10. M2c macrophages are identified by CD163 expression and associated with anti-inflammatory and apoptotic cell debris clearing functions by secreting high levels of TGF-β and IL-10 [26,44]. Finally, M2d macrophages, or tumor-associated M2, produce elevated quantities of IL-10, but low amounts of IL-12 and TGF-β. Cells can be differentiated by the presence of VEGF and IL-10 markers [42]. This subtype of macrophages is notorious for being a major inflammatory cell of a tumor microenvironment, where they are responsible for angiogenesis and metastasis [45,46].

M1 and M2 macrophages have different metabolic preferences [47,48]. In particular, M1 macrophages rely mainly on glycolysis for energy production, while their tricarboxylic acid (TCA) cycle is impaired and oxidative phosphorylation is reduced. In contrast, M2 macrophages are characterized by high levels of oxidative phosphorylation. There are multiple discrepancies in other metabolic pathways, namely, pentose phosphate shunt, fatty acid synthesis and oxidation, arginine and glutathione metabolism and others [47,48].

There are several controversies regarding the concept of macrophage polarization. The first is related to the nomenclature. Specifically, in the scientific literature, the terms “classically activated macrophages” and “alternatively activated macrophages” are very frequently used interchangeably with M1 macrophages and M2 macrophages, respectively. Nevertheless, originally the two classification schemes, i.e., classically activated/alternatively activated and M1/M2 polarized, were designed separately and possessed different meanings. Namely, classically activated macrophages and alternatively activated macrophages were defined as those activated by IFN-γ and IL-4, respectively, whereas M1/M2 macrophages were defined on the basis of their different response to bacterial LPS–M1 produce nitric oxide (NO) through inducible nitric oxide synthase (iNOS) expression and M2 synthesize ornithine through arginase expression [49]. Recently, Orecchioni and colleagues demonstrated that there is a significant discrepancy between the gene signatures of classically activated macrophages and M1 macrophages, as well as between alternatively activated macrophages and M2 [49]. For instance, classically activated macrophages exclusively expressed genes related to chemotaxis and cell migration, while only M1 macrophages expressed genes associated with anti-bacterial response. Similarly, alternatively activated macrophages solely expressed IL-4-induced standard cell surface markers, while M2 macrophages expressed genes that function in arginine and lipid catabolism. This suggests that the names classically activated/alternatively activated macrophages are not equivalent to the terms M1/M2 macrophages. The second controversy regarding macrophage polarization is related to macrophage plasticity. It is frequently reported in the literature that M1/M2 polarization is reversible due to macrophage plasticity. Thus, it is believed that M2 macrophages can be transdifferentiated towards a M1 phenotype by M1-polarizing factors and vice versa [26,50]. In fact, using a global transcriptomic profile, Liu and colleagues recently demonstrated that M1 and M2 macrophages could freely repolarize between the two phenotypes and did not retain memory from previous polarization states [51]. There are, however, certain disagreements regarding this conception. In particular, computer simulations of M1 and M2 gene regulatory networks that were built on the basis of published data showed that pro-inflammatory macrophages were unable to shift to an anti-inflammatory phenotype [52]. Thirdly, a seemingly clear classification of macrophages into two distinct polarization states, classically and alternatively activated, or M1 and M2, is probably an oversimplification since there are multiple phenotypes of macrophages with a significant overlap between the markers of each polarization state [53,54]. Novel high-content and high-throughput methods could potentially improve macrophage identification and characterization [55]. Several other controversies related to macrophage polarization can be found in the article by Dr. Peter Murray [56].

## 3. The Role of M1 and M2 Macrophages in MI

The polarization of macrophages in different settings plays a crucial role in tissue regeneration and the wound healing process [29]. Albeit the complex molecular mechanisms of polarization are yet to be determined, the topic provides an opportunity to find novel treatment methods for a number of diseases that require restoration of damaged tissues and organs [57,58,59]. MI is one of the diseases in which regulation of macrophage polarization could be beneficial [60]. Nevertheless, the macrophage profile in healthy and infarcted hearts are much more complicated than merely M1 and M2 polarization states [61]. In this section we will discuss the phenotypes of macrophages found in healthy as well as in infarcted cardiac tissue.

Similar to other tissues, the heart also contains resident macrophages [62]. Mouse models show that cardiac resident macrophages account for 6–8% of non-cardiomyocytes and arise from two distinct lineages—one is derived from erythromyeloid progenitors in the yolk sac during early embryogenesis, and the other originates from fetal monocytes in the postnatal period [63,64]. These two sets of macrophages differentiate from one another based on the expression of CCR2. Thus, yolk sac-derived macrophages are CCR2-, whereas monocyte-derived macrophages are CCR2+ [64]. CCR2- macrophages are mainly found in the myocardium of the heart and are replenished by self-renewal, while CCR2+ macrophages reside mostly in the endocardium and are restored by the recruitment of circulating monocytes [19,65]. CCR2- macrophages are further classified into three subsets based on the MHC-II and Ly6C expression—MHC-II^high^, MHC-II^low^ and Ly6C+ [11]. Overall, based on the outlined surface markers, resident cardiac macrophages can be categorized into four subsets, i.e., CCR2-MHC-II^high^, CCR2-MHC-II^low^, CCR2- Ly6C+ and CCR2+ macrophages. Recently, however, a study by Dick and colleagues has provided a slightly alternative description of the phenotypes of resident cardiac macrophages [17]. Using genetic fate mapping, long-term parabiosis studies and single-cell RNA sequencing in mice, the authors identified four subsets of macrophages in an adult heart—TIMD4+LYVE1+MHC-II^low^CCR2– macrophages, TIMD4−LYVE1–MHC-II^high^CCR2− macrophages and two CCR2+MHC-II^high^ subsets. It was shown that TIMD4+LYVE1+MHC-II^low^CCR2– macrophages are maintained by local proliferation independent from circulating monocytes, whereas TIMD4−LYVE1–MHC-II^high^CCR2− macrophages could be partially replaced by circulating monocytes, while CCR2+MHC-II^high^ subsets are restored exclusively by blood monocytes [17]. Although there is some evidence that questions the aforementioned ontogenic categorizations of resident cardiac macrophages, most of the data agree on the fact that only a small proportion of macrophages in the heart is derived from circulating monocytes [19]. Studies show that the function of cardiac resident macrophages is not restricted to protection but are also involved in the regulation of electrical conduction [66]. Besides conventional macrophages which express only macrophage-related markers, a healthy murine heart also contains hybrid cells which possess molecular signatures of macrophages and fibroblasts [67].

Early studies reported that the resident cardiac macrophages possessed an M2-like phenotype and expressed multiple M2 markers [16]. Nevertheless, an attempt to classify resident cardiac macrophages into distinct M1 and M2 categories is likely an oversimplification since macrophages possess a versatile plasticity and can dynamically change their surface markers [68]. There are several explanations regarding changes in the phenotypic profile of cardiac macrophages after MI. One established theory is that following the death of cardiac muscle, the heart becomes rapidly infiltrated with circulating Ly6C^high^ monocytes which differentiate into M1 macrophages [64,69]. This process is regulated by resident cardiac macrophages. In particular, CCR2+ macrophages promote monocyte recruitment via myeloid differentiation primary response 88 (MYD88) pathway [18]. Ly6C^high^ monocytes become the predominant cell type in the first few days post-MI and their number peaks at around day 3 after infarction [70]. M1 macrophages formed from monocytes engulf cellular debris, degrade ECM and amplify inflammation by secreting pro-inflammatory cytokines such as TNF-α, IL-1β and IL-6 [69]. A recent study by Liu and colleagues showed that M1-like pro-inflammatory macrophages also contributed to myocardial injury by secreting pro-inflammatory exosomes and pro-inflammatory miRNAs which inhibited angiogenesis and cardiac healing [71]. By day 4–7 after MI, another type of monocyte, namely, Ly6C^low^ monocytes become preferentially recruited to the infarcted myocardium. Ly6C^low^ monocytes give rise to M2 macrophages which suppress inflammation by IL-10 secretion and initiate ECM remodeling and angiogenesis [69]. This switch to an M2 phenotype could potentially be regulated by neutrophils since neutrophil-depleted mice had increased numbers of M2 macrophages [72]. There are, however, some controversies over this theory. In particular, it remains disputable whether M1 and M2 macrophages arise from distinct monocyte populations, i.e., Ly6C^high^ and Ly6C^low^ monocytes, respectively, or whether M1 macrophages can switch their phenotype to M2 [73]. Some studies, for instance, demonstrated that these Ly6C^low^ monocytes were actually Ly6C^low^ macrophages which originated from Ly6C^high^ monocytes [63]. Furthermore, as stated earlier, it is likely an oversimplification to categorize macrophages into only M1 and M2 phenotypes since there could be multiple phenotypes in between. This was shown in a study by Mouton and colleagues, in which cardiac macrophage transcriptomic signatures were analyzed on days 1, 3 and 7 after MI in mice [74]. Although macrophages on day 1 showed a profound pro-inflammatory phenotype, whereas macrophages on day 7 expressed a reparative phenotype, both types did not exclusively express the markers of either M1 or M2. For instance, day 1 macrophages overexpressed Arg1, which is considered a classical M2 marker. Moreover, the expression of IL-10 did not significantly increase from day 3 to day 7, which is predicted by the M1/M2 theory. These observations supported the idea that macrophages in an infarcted heart should not be viewed in the context of two distinct M1 and M2 polarization states but rather considered on the continuum of phenotypes [74]. In summary, in the first few days after MI, macrophages with an M1-like pro-inflammatory phenotype predominate in the infarcted myocardium, while at later stages, macrophages with M2 anti-inflammatory properties become more prevalent. Nevertheless, their phenotypes should not be reduced to simply M1 and M2, since there is a great heterogeneity in the cardiac macrophage population, both in a healthy, as well as an infarcted heart.

## 4. Therapeutic Implications

Following MI, the macrophage profile changes. In the first few days post-MI, the injured heart is dominated by pro-inflammatory macrophages, whereas several days later, macrophages with the anti-inflammatory phenotype take over [74,75]. Several studies have shown that altering the macrophage profile of the infarcted heart by stimulation towards M2-like phenotype could be beneficial for cardiac tissue repair [76]. In this section, we will review various strategies used to promote M2 polarization in the acute MI settings as well as their therapeutic implications.

### 4.1. Cytokines, Bioactive Molecules and Drugs

Cytokines and other bioactive molecules have been utilized to induce M2 phenotype in the infarcted heart. In a study by Shintani and colleagues, the long-acting form of IL-4 was applied to induce the alternative activation of macrophages and improve their function in post-MI mice [77]. The treatment led to a significant increase in the amount of CD206+F4/80+ M2-like macrophages. Moreover, the hearts treated with IL-4 experienced enhanced cardiac repair, improved cardiac function and alleviated negative ventricular remodeling. The latter was shown to be attributed to the activity of M2 macrophages rather than to the direct action of IL-4. Specifically, the application of IL-4 to the mice that were unable to form M2 macrophages could not attain the aforementioned positive effects. Interestingly, the beneficial effects of IL-4 on macrophage polarization, as well as heart regeneration, were time-dependent, i.e., it was not possible to attain the effects 28 days after MI. Furthermore, it was shown that IL-10 can induce M2 polarization [78]. Jung and colleagues described the effects of the anti-inflammatory cytokine in the regulation of the balance between macrophage populations. Results showed that the infusion of IL-10 activated the polarization of the M2 macrophages without any effect on the M1 subtype in a mouse model of MI [78]. Additional effects included proliferation and migration of the fibroblasts and reduction of ECM. Overall, IL-10 increased the population of M2 macrophages, reduced inflammation in the left ventricle and improved wound healing. Inhibition of IL-6 could also stimulate M2 macrophages in a mouse MI model. In the study by Jing and colleagues, IL-6 knock-out mice had considerably higher numbers of M2 macrophages, better cardiac functions and greater 28-day survival after MI compared to wild type mice [79].

Plasmin is another molecule involved in M2 macrophage polarization. Carlson and colleagues reported that the macrophage polarization could be mediated not only via urokinase plasminogen activator (uPA) but via an alternative pathway as well. The authors demonstrated that mice deficient in uPA still developed M2 polarization [80]. Inhibition of DNAX accessory molecule 1 (DNAM-1), or CD226 receptor, is another strategy that can be utilized for the therapeutic activation of M2 following MI. CD226 expression on macrophages is highly increased in post-infarction heart tissue and CD226 knockout mice had better recovery after left anterior descending artery ligation [81]. Moreover, CD226 deletion tended to alleviate M2 macrophage polarization, while suppressing M1 type cells as well as creating a reparative healing microenvironment [81]. Angiotensin II (Ang II) can also be utilized to induce M2 phenotype in the infarcted hearts. Liu and colleagues discovered that Ang II indirectly promoted M2 polarization in mice with MI by enhancing the expression of thymic stromal lymphopoietin (TSLP) in macrophages, cardiac fibroblasts and vascular smooth muscle cells [82]. TSLP is a cytokine with anti-inflammatory effects that is known to stimulate macrophages towards the M2 phenotype. Hydrogen sulfide (H_2_S) is yet another chemical that was shown to induce M2 polarization [83]. H_2_S is endogenously produced in the human body agent and participates in multiple physiologic processes and diseases [84]. In particular, it exhibits cardioprotective effects in the infarcted hearts [85]. Miao and colleagues showed that these protective effects were partially mediated by M2 macrophage activation [83]. Thus, the authors created mice that were deficient in an enzyme involved in H_2_S generation and observed their convalescent state after MI. The mice had poorer survival and worsened cardiac function. By contrast, wild type mice and enzyme-deficient mice that received H_2_S donor supplementation had improved cardiac function and remodeling, as well as higher survival rates. The authors also proposed that the M2 activation by H_2_S was potentially mediated by increased mitochondrial generation of lipolysis and fatty acid oxidation.

ECM components can also be utilized for activation of the M2 phenotype. In their recent study, Wang and colleagues used hyaluronic acid-derived short oligosaccharides (HA-o) for wound healing after MI [86]. The authors reported that HA-o improved the macrophage polarization towards M2 phenotype in vitro as well as decreased the infarct size, reduced apoptosis and stimulated angiogenesis in a mouse model. In another study, recombinant collagen type I and type III matrices were used for MI treatment in a mouse model [87]. The treatment caused a 1.5-fold increase of M2 macrophages 28 days post-MI. Furthermore, there was an enhanced cardiomyocyte survival and angiogenesis but reduced scar formation.

Certain plant-derived bioactive molecules were also demonstrated to promote alternative macrophage polarization. A recent study showed that N-propargyl caffeate amide (PACA), a caffeic acid derivative, could increase the expression of M2 markers while reducing the expression M1 markers both in vitro and in a rat model of MI [88]. The process was likely to be mediated via the peroxisome proliferator-activated receptor-gamma (PPAR-γ) pathway. Resveratrol, a plant-derived bioactive molecule with anti-inflammatory and antioxidative properties, is another compound that was shown to drive the M2 polarization in a mouse model of MI [89,90].

Drug carriers and certain medications have been shown to enhance the quantity of M2 macrophages in the infarcted myocardium. Torrieri and colleagues intended to use macrophage hitchhiking in order to target acetalated dextran-based nanoparticles containing cardiomyocyte-stimulating compounds to the infarcted heart [91]. For this purpose, linear TT1, a special peptide that can be internalized by macrophages associated with atherosclerotic plaques, was attached to the nanoparticles. The authors discovered that the peptide was incorporated to a greater extent by M2 macrophages compared to M1, which was evidenced for both murine and human macrophages. This suggests that nanoparticles containing linear TT1 can be utilized to preferentially recruit M2 macrophages to the infarcted heart. In the study by Ben-Mordechai and colleagues, hyaluronan lipid-based particles containing hemin, an enzyme that activates heme oxygenase-1, were used to target infarct macrophages [92]. The particles induced M2 phenotype expression in infarct macrophages and attenuated adverse ventricular remodeling. Certain medications can also be used to promote M2 polarization. Sodium-glucose cotransporter 2 (SGLT2) inhibitors are one class of such medications. In the study by Lee and colleagues, an SGLT2 inhibitor dapagliflozin was tested for its ability to enhance the alternative polarization of macrophages in a rat MI model [93]. It was found that the drug effectively increased the number of M2 macrophages while reducing the number of M1 macrophages. Moreover, the infarcted hearts treated with dapagliflozin showed a smaller number of myofibroblasts and decreased fibrosis several weeks after the infarction. The authors showed that the effects of dapagliflozin were mediated via the signal transducer and activator of transcription 3 (STAT3) signaling pathway. Importantly, the same beneficial effects were achieved by phlorizin, a non-specific SGLT1/2 inhibitor, but to a much smaller extent. 

### 4.2. Regulation of the Immune System

Regulation of the immune system is another alternative for the enhancement of M2 polarization. Several studies have reported that regulatory T cells (T regs) are critical regulators of the M1 and M2 balance [94,95]. Choo and colleagues used tolerogenic dendritic cells to activate Tregs in a mouse MI model [96]. The treatment caused a rapid switch of M1 macrophages to M2 phenotype. By contrast, the non-treated mice still had a high number of M1 macrophages in their hearts. Another strategy for alternative macrophage polarization is the suppression of signaling pathways involved in immune cell activation. Tokutome and colleagues demonstrated that inhibition of nuclear factor-κB (NF-κB) could increase alternative macrophage activation. The researchers used pioglitazone loaded into poly (lactic acid/glycolic acid) nanoparticles (NPs) to target peroxisome proliferator-activated receptor-gamma (PPARγ) for NF-κB inhibition. This resulted in enhanced M2 polarization and improved cardiac repair in the mouse MI model [97]. In a study by Li and colleagues, knock-out of CD226, a receptor constitutively expressed on T cells, NK cells and monocytes, and involved in cell-mediated cytotoxicity, resulted in increased F4/80+ CD206+ M2 macrophages and decreased Mac-3+ iNOS+ M1 macrophages in mice with MI [81].

It is important to mention that immune cells have different effects on macrophage polarization in immature hearts compared to adult cardiac tissue. Thus, it was generally accepted that T regs promote M2 phenotypes in an adult heart [98]. However, recently, Li and colleagues demonstrated that in neonatal hearts, T regs suppress the activation of M2 macrophages and inhibit fibrosis [99]. CD4+ T cells are another population of cells with regulatory properties and distinct cytokine expression patterns [100]. In particular, in a recent study, the ablation of CD4+ T cells led to the decline of M2 macrophages in a juvenile heart but did not have a significant effect on the quantity of M1 or M2 macrophages in an adult heart [101]. This important aspect should be taken into account when immune cells are planned to be utilized as a therapeutic strategy for the control of macrophage polarization.

### 4.3. Stem Cells and Exosomes

Stem cells (SC) and SC-derived exosomes are other strategies to induce M2 phenotype. Recently, it was reported that pro-inflammatory macrophages could enhance the therapeutic potential of mesenchymal stem cells (MSCs) in tissue regeneration [102]. This beneficial effect can be obtained in a reverse direction as well, i.e., MSCs can act on macrophages to improve tissue repair. Human umbilical cord blood-derived mesenchymal stem cells were shown to stimulate the M2 phenotype inside the heart as well as in the extracardiac tissues such as peripheral blood and spleen in the acute MI model of mice [103]. Lee and colleagues demonstrated that n-butylidenephthalide-preconditioned adipose-derived stem cells (ADSCs) stimulated M2 polarization and reduced cardiac fibrosis via PI3K-STAT3 pathway in rats with MI [104]. In the study by Deng and colleagues, exosomes generated from adipose-derived mesenchymal stem cells were found to promote M2 phenotype in the animal model of MI [105]. The authors also reported that the effect was mediated via the S1P/SK1/S1PR1 signaling pathway, which is consistent with data published by Cho and colleagues [106]. Thus, MSCs injection in a rat model of MI significantly reduced the expression of M1 markers, such as IL-6, IL-1β, monocyte chemoattractant protein-1 and iNOS. In contrast, the expression of M2 markers such as IL-10, IL-4, CD206 and Arg1, showed a significant elevation. One of the ways MSCs could mediate M2 polarization is through angiopoietin-like 4 (ANGPTL4) protein. Cho and colleagues demonstrated that ANGPTL4-deficient mice had an overexpression of pro-inflammatory markers though a weak expression of anti-inflammatory molecules [107]. Recently, Zhang and colleagues demonstrated that transplantation of CD146+ MSCs have superior effects on macrophage polarization in a mouse MI model [108]. Mouse bone marrow MSCs derived exosomes promoted in vitro and in vivo polarization of M1 to M2 via delivering miR-182 that attenuated TLR4 through TLR4/NF-κB/PI3K/Akt pathway. Exosomes also enhanced the recovery of myocardial ischemia/reperfusion (I/R) mice by reducing infarction size and inflammation [109]. Exosomes derived from lipopolysaccharide-pre-conditioned bone marrow-derived mesenchymal stem cells (BMSCs) were shown to be superior in polarizing macrophages to M2 phenotype in a mouse MI model compared to exosomes derived from conventional BMSCs [110]. The effect was mediated by the NF-κB signaling pathway inhibition and partial activation of the AKT1/ AKT2 signaling pathway. Exosomes derived from regulatory T cells could reduce the expression of M1-specific proteins but enhance the expression of M2 markers in mice with MI [111]. Table 1 summarizes approaches that are used to enhance M2 polarization in MI models. Overall, multiple approaches such as cytokine and bioactive molecules, drugs, immune system regulation, exosomes and stem cells, and others have been successfully utilized in animal models in order to enhance M2 polarization of macrophages after MI. These strategies can potentially become efficient therapeutic strategies for the treatment of MI.

## 5. Conclusions

Along with other cardiovascular diseases, myocardial infarction (MI) continues to pose a significant socio-economic burden worldwide. The development of new therapeutic strategies for MI treatment requires a thorough understanding of the pathogenesis of the disease. The cells of the innate immune system and macrophages, in particular, play an important role in the pathogenesis of MI. Cardiac resident macrophages, as well as monocyte-derived macrophages, are involved in the inflammation initiation, progression and resolution following MI. These processes are mediated by various phenotypes of cardiac macrophages, which were analyzed in this paper in terms of M1 and M2 phenotypes. Although the infarcted heart contains subsets of macrophages, which have some similarities to M1 and M2 macrophages in terms of expression markers and functions, the cardiac macrophage profile is much more complicated and dynamic. Nevertheless, we used this classification scheme to review some therapeutic options of macrophage polarization for MI therapy. Numerous strategies, such as cytokines and bioactive molecules, drugs and drug carriers, immune system regulation, exosomes and stem cells and others, were shown to stimulate M2 macrophage polarization and improve cardiac tissue repair in MI models. Despite the considerable number of promising results, several important aspects should be considered when this therapeutic approach is translated to clinical trials, namely, the complexity and heterogeneity of the macrophage population in the heart, the difference between human macrophages and macrophages from animal models, the need for an urgent treatment following MI and many others.

## Figures and Tables

**Table 1:** Approaches that are used to enhance M2 polarization in MI models.

Approach	Treatment	M2 Macrophage Phenotype	Reference
Cytokine treatment	IL-4	CD206+F4/80+	[77]
	IL-10	Arg1+Mrc1+Tgfb1+Ym1+ Fizz-1+	[78]
	IL-6	CD206+	[79]
Bioactive molecule treatment	Plasmin	Arg1+Ym1+Fizz-1+	[80]
	Angiotensin II	CD206+	[82]
	Hydrogen sulfide	CD206+F4/80+	[83]
	Hyaluronic acid-derived short oligosaccharides	CD206+	[86]
	Collagen type I and type III matrices	CD206+MMP1+Arg1+	[87]
Plant-derived bioactive molecules	N-propargyl caffeate amide	M2a: CD163+FZZ1+YM-1+IL-10+Arg1+	[88]
	Resveratrol	CD206+F4/80+	[90]
Lipid particles with a drug	Hyaluronan lipid-based particles containing hemin	CD206+F4/80+	[92]
Drug	SGLT2 Inhibitor Dapagliflozin	CD206+F4/80+CD68+IL-10+	[93]
Regulation of the immune system	Activation of Treg by tolerogenic dendritic cells	CD68+MR+	[96]
	NF-κB inhibition by pioglitazone loaded into poly (lactic acid/glycolic acid) nanoparticles	Not specified	[97]
	Knock-out of CD226	CD206+F4/80+	[81]
Stem cell therapy	Intravenous transplantation of hUCB-MSCs	CD11b+Ly6C- and F4/80+iNOS-	[103]
	CD146+ MSCs	CD163+F4/80+	[108]
Exosomes	ADMSCs-derived exosomes	CD206+	[105]
	Mesenchymal stromal cell-derived exosomes	CD206+ iNOS-	[109]
	Exosomes derived from LPS-pre-conditioned BMSCs	CD206+ArgI+	[110]
	Treg-derived exosomes	CD206+F4/80+Arg-1+TGF-β+	[111]
